# Evaluation of the Nutritive and Organoleptic Values of Food Products Developed by Incorporated *Catharanthus roseus* (*Sadabahar*) Fresh Leaves Explore Their Hypoglycemic Potential

**DOI:** 10.1155/2014/304120

**Published:** 2014-03-24

**Authors:** Gita Bisla, Shailza Choudhary, Vijeta Chaudhary

**Affiliations:** ^1^Department of Food Science and Nutrition, Banasthali University, Rajasthan 304022, India; ^2^Department of Commerce, V.M.L.G College, Ghazibad, Uttar Pradesh, India

## Abstract

Diabetes becomes a real problem of public health in developing countries, where its prevalence is increasing steadily. Diabetes mellitus can be found in almost every population in the world. Since the Ayurvedic practice started in India, plants are being used in the cure of diseases. Although the *Catharanthus roseus* have been used for their alleged health benefits and avail their hypoglycemic effect, used as medicine by diabetics. Medicinal plants have rarely been incorporated in food preparations. To fill these lacunae, food products were prepared by using *Catharanthus roseus* (*Sadabahar*) fresh leaves with hypoglycemic properties. Commonly consumed recipes in India are prepared for diabetic patients and were developed at different levels at 3 g, 4 g, and 6 g per serving. Food product development and their acceptability appraisal through organoleptic evaluation were carried out by semitrained panel comprising 15 trained panelists from the department of Food Science and Nutrition, Banasthali University. Seven products were developed by incorporating *Catharanthus roseus* fresh leaves. Nine point hedonic scale was used as a medium to know about the product acceptability at various variances. All products are moderately acceptable at different concentrations except product fare “6 g” which was more acceptable than the standard. Among the three variations of incorporating the *Catharanthus roseus* (*Sadabahar*) Leaves, 3 g variation is more acceptable than other variations.

## 1. Introduction

Diabetes mellitus is a chronic condition characterized by major derangements in glucose metabolism and abnormalities in fat and protein metabolism [1]. Patients use many products and modalities to treat diabetes or its comorbidities. People with diabetes are 1.6 times more likely to use a complementary and alternative medicine (CAM) treatment modality than those without diabetes [2].

The World Health Organization (WHO) estimates that currently more than 180 million people worldwide have diabetes and it is likely to double by 2030, with India, China, and the United States predicted to have the largest number of affected individuals [3]. Many plants and their active chemical compounds have demonstrated activity in the treatment of various disorders [4]. According to ethnobotanical information, more than 800 plants are used as traditional remedies in one or other form for the treatment of diabetes [5]. With the disturbing rise in the prevalence of this metabolic disease and associated healthcare cost, interest in alternative or complementary therapies has been grown. This interest is due to reasons such as ease of access, better culture acceptability and compatibility, cost effectiveness, and also the bid to “Go Natural”.

A plant is a living organism able to convert inorganic material into organic molecules necessary for the life of the plant itself and serves as food, for example, insects, animals, and humans. Plants also provide medicines and many other commodities. However, our knowledge about plants with their enormous diversity is still limited in many aspects. Still many novel products might be obtained from plants; however, this is hampered by the rapid loss of plant diversity on earth due to, for example, deforestation. The sustainable exploitation of plants for food and medicines requires extensive knowledge about plants. As the world's population grows to an estimated 9 billion people in 2050, the availability of food and medicines for all people in the future should be a concern to all of us. Up to now, plants remain a primary source of medicines for most people in the world. Therefore, research in plant science is of great importance for human health, both for the production of healthier food and for development and production of medicines [6].

The important values of some plants have long been published but a large number of them remain unexplored. So, there is a necessity to explore their uses and to conduct pharmacognostic and pharmacological studies to ascertain their therapeutic properties. Despite considerable progress in the management of diabetes mellitus by synthetic drugs, the search for natural antidiabetic plant products for controlling diabetes is going on. There are many hypoglycemic plants known through the folklore but their introduction into the modern therapy system awaits the discovery of animal test system that is closely parallel to the pathological course of diabetes in human beings. Hypoglycemic activity has been reported in many plants during the last twenty years [7].

In India, herbal medicines date back several thousand years to the Rig-Veda, a collection of Hindu sacred verses, system of health care known as Ayurvedic medicine, which is still widely practiced in India today.* Catharanthus roseus* belongs to the family apocynaceae [8]* Catharanthus roseus* is commonly called as* Periwinkle*,* Madagascar periwinkle*, and* Sadabahar*. It grows throughout India and is found as an escape in waste places and sandy tracts. More than 130 different compounds have been reported including about 100 monoterpenoid indole alkaloids [9]. As an important medical plant, it has a good antioxidant potential throughout its parts under drought stress [10]. There are several health benefits of* Catharanthus roseus* leaves such as maintaining blood sugar [11], lowering high blood pressure [12], menstruation irregularities, Hodgkin's disease [13], and as antioxidant [14].

Recently there has been a shift in universal trend from synthetic to herbal medicine, which we can say “Return to Nature.” Subsequently, with this background in mind, this study was embarking on the study of hypoglycemic effect of* Catharanthus roseus* fresh leaves in type II Diabetes Mellitus. Most of the people do not know* Catharanthus roseus* nutritional quality, health benefits, and how to use and what to use. They are not utilized to its full potential. So, the basic aim of this study is to create awareness about* Catharanthus roseus* leaves among the population. As many other herbal plants established in the indigenous system of medicine for their antidiabetic potentials and this plant has to be established in this indigenous system of medicines. Therefore, the present study was undertaken to develop commonly consumed food preparations for diabetics by incorporating* Catharanthus roseus* fresh leaves and evaluate their acceptability. Traditional foods reflect cultural inheritance and have left their imprints on contemporary dietary patterns [15].

## 2. Material and Methods

### 2.1. Sample Description

*Catharanthus roseus* fresh leaves were collected from the main campus of Banasthali University, Rajasthan, India. The leaves were washed thoroughly with tap water followed with sterilized distilled water for the removal of dust and sand particles. Commonly seven consumed recipes in India are prepared for diabetic patients were developed at different levels at 3 g, 4 g, and 6 g per serving.

### 2.2. Panel Selection and Training

Food product development and their acceptability appraisal through organoleptic evaluation carried out by a trained panel comprising 30 panelists from the department of Food Science and Nutrition, Banasthali University. They were preselected on the basis of good health conditions, time availability, no allergy plants products, any aversion to* Catharanthus roseus*, and willingness to participate. Panelists were then subjected to preliminary acuity tests to investigate their ability to recognize basic taste, basic aromas, and to describe basic attributes in prepared products by triangle method [16, 17]. After the screening process fifteen panelists were selected. Twelve panelists were female and three were male in the range of 20–35 years old of age. They went under training to perform the sensory evaluation of the products.

Training consisted of: (1) initial orientation session where panelists received detailed explanation about the organoleptic methodology; (2) group meetings for general description of the* Catharanthus roseus* fresh leaves incorporating food products development, which required 30 minutes to 1-hour sessions; (3) individual training during opening sessions panelists, took part in exercise. They were provided with the three samples (plain, salted, and sugary flavors in mango shake), which were diluted in 2% of cinnamon solution. They were asked to smell and taste them and list as many aromas and flavor terms as possible for each sample and discuss individual results to come up with a consensus.

### 2.3. Outcome Measures

The panelists performed organoleptic appraisal by 9 point hedonic test [18] to assess the overall products preferences. Evaluation of taste, flavor, color, texture, appearance, after taste, and overall acceptability were made in the scale. Two samples were during a 60-minute session and the evaluations were repeated two more times and they were asked to rinse their penchant well with water between samples. For this purpose, panelists used room-temperature drinking water.

### 2.4. Quality Control

- Panelist should be fit and free from tiredness to reduce the possibility of errors.
- Ensure that there is sufficient light at the judgment site.
- If too many people congregate, it may interfere with the sensory evaluation, site should be free from distraction.
- Drink water between the evaluations of samples.
- The panelists were instructed not to swallow the samples.
- Proper weighing of the raw materials to be used in the cooking.
- Area should be odor free.
- There should be proper ventilation.
- Instruction to the panelists should be understandable, concise, and appropriate to the test.

### 2.5. Standardization of Food Products and Nutritive Value of Food Products

Fresh leaves of medicinal plants, namely,* Catharanthus roseus*, leaves incorporating in food products, respectively, developed different levels at 3 g, 4 g, and 6 g per serving. Food preparations made by using different cooking methods (Table 1). Nutrient content per serving of the products incorporated* Catharanthus roseus* fresh leaves was calculated from the values given in nutritive value of Indian foods [19].

### 2.6. Statistical Analysis

The data were subjected to statistical analysis using Statistical Package for Social Sciences (SPSS) version 16.0. Mean ± SD was used to obtain the differences in organoleptic scores, within different levels of incorporation of* Catharanthus roseus* fresh leaves in food preparations. Level of significance was accepted at *P* ≤ 0.05.

## 3. Results

### 3.1. Organoleptic Evaluation

All the food products incorporated with* Catharanthus roseus* (*Sadabahar*) fresh leaves were found to be organoleptically satisfactory at different variations. Comparing the food products at different variations with standard of the food products, respectively. Although the scores of standard was more acceptable than all the variations made at different concentrations. It was also observed that the concentration of* Catharanthus roseus *fresh leaves in the samples was inversely proportional to the acceptability scores except in product* fure* where the scores of “6 g” were better than all the variations made at different concentrations for all attributes. As the concentration was increased, the mean scores for appearance, Color, flavor, texture, taste, after taste, and overall acceptability were found to be increased.

In* Catharanthus roseus* (*Sadabahar*) fresh leaves incorporated food products (Table 2), namely,* fure, *the most acceptable level of incorporation was 6 g and the mean scores for overall acceptability were 8.63 ± 0.82. However, scores for all organoleptic characteristics of* fure* within 3 g and 4 g levels of incorporation were found to be nonsignificantly different.

*Rasam* and* Stuffed Idli* were moderately acceptable and the scores for their overall acceptability awarded by the panel of judges were 7.0 ± 1.23 at 3 g and 7.4 ± 0.06 at 4 g, respectively. Nonsignificant difference was noticed in scores for all organoleptic characteristics of* Rasam* and* Stuffed Idli* within variations of incorporation of* Catharanthus roseus* (*Sadabahar*) fresh leaves (Table 2). However, the acceptable level of incorporation varied in different food products. It has been found that when the level of incorporation of fresh leaves increased beyond the accepted levels in any food products,the mean scores for all organoleptic characteristics decreased.

For* palak methi muthia*,* methi parantha*,* coriander chutney*, and* cucumber soup* the most acceptable level of incorporation of* Catharanthus roseus* (*Sadabahar*) fresh leaves was 3 g per serving and the respective scores for overall acceptability ranged from 6.6 ± 0.07 (*palak methi muthia*) to 7.15 ± 0.09 (*cucumber soup*). Nonsignificant differences were found in scores for all organoleptic characteristics of food products (Table 2).

### 3.2. Nutritive Evaluation

Energy content of all the food products prepared by incorporating 3 g* Catharanthus roseus* (*Sadabahar*) fresh leaves per serving was varied from 13.31 Kcal of* cucumber soup* to 240.34 Kcal per serving of* methi parantha* (Table 3). Protein content was the highest in* fure *(12.51 g/serving) and the lowest in* cucumber soup* (0.34 g/serving). Fat content ranged between 0.03 g in* cucumber soup* and 12.51 g per serving in* fure*. Carbohydrate content of 3 g* Catharanthus roseus* (*Sadabahar*) fresh incorporated food products varied from 1.18 g of soup to 35.66 g per serving of* stuffed idli*. The highest fibre content was found in* coriander chutney* (0.75 g/serving) and minimum in soup was (0.05 g/serving). The hypoglycemic activity of* Catharanthus roseus* leaves may be due to the presence of alkaloid-like vincristine and vinblastine through stimulation of *β* cell activity leading to increased insulin production and release [20].

## 4. Conclusions

The scores of standard were more acceptable than all the variations made at different concentrations. It was also observed that the concentration of* Catharanthus roseus* fresh leaves, in the samples was inversely proportional to the acceptability scores. Three g per serving* Catharanthus roseus* freshleaves incorporation in food products developed was found to be organoleptically acceptable by the semitrained panel members. Amongst the incorporated food preparations, the mean scores for overall acceptability were highest for* fure* and lowest for methi parantha. Thus, this holds great promise for future research for the formulation of potent antidiabetic drug for the present plant.

## Figures and Tables

**Table 1:** Food preparations prepared with different methods of cooking by incorporating *Catharanthus roseus* fresh leaves.

Food preparations	Method of processing
Fure	Steaming
Rasam	Roasting and pressure cooking
Stuffed idli	Fermentation and steaming
Palak methi muthia	Steaming
Methi paratha	Stir frying
Coriander chutney	Grinding
Cucumber soup	Pressure cooking

**Table 2:** Organoleptic evaluation of food products developed by incorporation of *Catharanthus roseus* fresh leaves.

Food products	g/serving	Appearance	Color	Texture	After taste	Overall acceptability
Fure	Standard	8.2 ± 0.08	8.5 ± 0.61	8.5 ± 0.56	8.3 ± 0.03	8.25 ± 0.52
	3 g	8.6 ± 0.82	8.06 ± 1.25	8.4 ± 0.61	8.2 ± 0.09	8.3 ± 0.87
	4 g	8.6 ± 0.89	8.7 ± 0.56	8.4 ± 1.06	8.4 ± 0.76	8.4 ± 0.06
	6 g	8.7 ± 0.05	8.8 ± 0.59	8.5 ± 0.56	8.5 ± 0.53	8.63 ± 0.82

Rasam	Standard	7.2 ± 0.06	8.1 ± 0.71	7.52 ± 0.67	8.3 ± 0.06	8.2 ± 0.56
	3 g	7.2 ± 0.08	7.4 ± 0.93	7.2 ± 1.10	7.5 ± 0.89	7.0 ± 1.23
	4 g	7.8 ± 0.32	7.0 ± 0.04	6.1 ± 0.58	6.3 ± 0.79	6.7 ± 0.15
	6 g	7.0 ± 0.85	7.1 ± 0.88	5.42 ± 1.23	5.6 ± 0.40	6.02 ± 0.04

Stuffed idli	Standard	8.0 ± 0.54	7.5 ± 0.02	7.5 ± 0.08	8.5 ± 0.79	8.0 ± 0.63
	3 g	7.6 ± 0.80	7.9 ± 0.56	7.49 ± 1.61	7.21 ± 0.43	7.3 ± 0.32
	4 g	7.20 ± 0.34	7.7 ± 0.12	7.4 ± 0.06	6.4 ± 0.11	7.4 ± 0.06
	6 g	6.69 ± 0.84	6.7 ± 0.65	6.5 ± 1.23	6.5 ± 0.49	6.62 ± 0.98

Palak methi muthia	Standard	8.0 ± 0.23	7.1 ± 0.99	8.1 ± 0.72	7.9 ± 0.88	8.05 ± 0.66
	3 g	7.8 ± 0.04	6.43 ± 0.99	7.3 ± 1.01	6.3 ± 0.61	6.6 ± 0.07
	4 g	6.2 ± 0.63	6.0 ± 0.70	6.7 ± 0.85	6.31 ± 0.56	6.1 ± 0.51
	6 g	6.06 ± 0.78	6.11 ± 0.44	5.07 ± 0.45	6.6 ± 0.09	6.0 ± 0.74

Methi parantha	Standard	8.85 ± 0.80	8.06 ± 0.18	8.50 ± 0.66	7.5 ± 1.23	8.01 ± 0.36
	3 g	7.69 ± 0.55	6.61 ± 0.74	6.45 ± 0.17	6.22 ± 0.99	6.36 ± 0.47
	4 g	6.09 ± 0.22	6.2 ± 0.50	5.49 ± 0.75	6.24 ± 1.06	6.01 ± 0.89
	6 g	5.43 ± 0.68	5.38 ± 0.34	5.5 ± 0.32	5.5 ± 0.53	5.6 ± 0.62

Coriander chutney	Standard	8.5 ± 0.54	8.81 ± 0.28	8.15 ± 0.01	8.05 ± 0.54	8.42 ± 0.33
	3 g	8.3 ± 0.14	8.24 ± 0.39	8.34 ± 0.09	8.08 ± 0.37	8.1 ± 0.67
	4 g	8.09 ± 0.54	7.8 ± 0.04	7.5 ± 0.87	7.27 ± 0.01	7.08 ± 0.69
	6 g	8.0 ± 0.87	7.04 ± 0.88	6.98 ± 0.59	6.62 ± 0.37	7.03 ± 0.03

Cucumber soup	Standard	7.76 ± 0.54	8.0 ± 0.77	7.5 ± 1.32	8.3 ± 0.98	8.12 ± 0.59
	3 g	7.6 ± 0.82	6.07 ± 0.25	7.4 ± 0.36	7.2 ± 0.09	7.15 ± 0.09
	4 g	6.87 ± 0.03	6.55 ± 1.87	6.08 ± 0.43	5.29 ± 0.26	6.28 ± 0.37
	6 g	6.6 ± 0.54	5.9 ± 0.66	5.05 ± 0.67	5.76 ± 0.67	5.54 ± 0.51

All values are found to be Nonsignificant.

**Table 3:** Nutritive evaluation of food products developed by incorporation of 3 g *Catharanthus roseus* fresh leaves per serving.

Food products	Cooked weight/serving (g)	Energy (kcal)	Protein (g)	Fat (g)	Carbohydrate (g)	Fiber (g)
Fure	80	137.34	12.51	3.87	34.58	0.16
Rasam	244	80.2	3.43	3.58	17.1	0.69
Stuffed idli	78	142.76	7.07	0.84	35.66	0.58
Palak methi muthia	65	168.52	10.58	2.65	23.04	0.12
Methi paratha	92	240.34	11.32	7.98	29.65	0.11
Coriander chutney	46	55	1.67	0.07	3.5	0.75
Cucumber soup	185	13.31	0.34	0.03	1.18	0.05
